# Th2 Cytokines Inhibit Lymphangiogenesis

**DOI:** 10.1371/journal.pone.0126908

**Published:** 2015-06-03

**Authors:** Ira L. Savetsky, Swapna Ghanta, Jason C. Gardenier, Jeremy S. Torrisi, Gabriela D. García Nores, Geoffrey E. Hespe, Matthew D. Nitti, Raghu P. Kataru, Babak J. Mehrara

**Affiliations:** The Department of Surgery, Division of Plastic and Reconstructive Surgery, Memorial Sloan Kettering Cancer Center, New York, New York, United States of America; University of South Florida, UNITED STATES

## Abstract

Lymphangiogenesis is the process by which new lymphatic vessels grow in response to pathologic stimuli such as wound healing, inflammation, and tumor metastasis. It is well-recognized that growth factors and cytokines regulate lymphangiogenesis by promoting or inhibiting lymphatic endothelial cell (LEC) proliferation, migration and differentiation. Our group has shown that the expression of T-helper 2 (Th2) cytokines is markedly increased in lymphedema, and that these cytokines inhibit lymphatic function by increasing fibrosis and promoting changes in the extracellular matrix. However, while the evidence supporting a role for T cells and Th2 cytokines as negative regulators of lymphatic function is clear, the direct effects of Th2 cytokines on isolated LECs remains poorly understood. Using *in vitro* and *in vivo* studies, we show that physiologic doses of interleukin-4 (IL-4) and interleukin-13 (IL-13) have profound anti-lymphangiogenic effects and potently impair LEC survival, proliferation, migration, and tubule formation. Inhibition of these cytokines with targeted monoclonal antibodies in the cornea suture model specifically increases inflammatory lymphangiogenesis without concomitant changes in angiogenesis. These findings suggest that manipulation of anti-lymphangiogenic pathways may represent a novel and potent means of improving lymphangiogenesis.

## Introduction

Lymphangiogenesis is the process by which new lymphatic vessels form from pre-existing lymphatic vessels in response to pathologic stimuli such as wound healing, inflammation, and tumor metastasis [1, 2]. This process is coordinated by a complex interplay between cytokines and growth factors that promote or inhibit lymphatic endothelial cell (LEC) proliferation, migration, and differentiation. A large number of studies focusing on lymphangiogenic cytokines, particularly within the vascular endothelial growth factor (VEGF) family, have elucidated critical roles for these molecules in regulating virtually every aspect of lymphatic vessel formation [3–5]. More recently, a few studies have reported on molecules that have anti-lymphangiogenic effects in some physiologic circumstances, including transforming growth factor beta-1 (TGF-β1), interferon gamma (IFNγ), and endostatin, among others [6–11]. Although the exact mechanism of how these cytokines and growth factors regulate lymphangiogenesis remains unclear, it has been suggested that these inhibitory pathways are homeostatic, aiming to control lymphangiogenesis [11]. It has also been suggested that different cell types promote (i.e. macrophages and B cells) or inhibit (i.e. T cells) lymphangiogenesis [11–13]. Understanding the complex interplay between cells and soluble growth factors in regulating lymphangiogenesis is critically important and has a wide scientific impact given the lymphatic system’s role in regulating inflammation, immunity, metabolism, and tumorigenesis.

Our group has recently reported that lymphedema, both clinically and in a mouse tail model, results in a significant inflammatory response [14, 15]. We have characterized this response by showing that T cells comprise nearly 70% of all inflammatory cells in chronic lymphedema and is associated with a mixed T-helper 1 (Th1) and T-helper 2 (Th2) response, which is in contrast to the inflammatory cell infiltrate observed in acute edema (i.e. surgical edema that resolves quickly and spontaneously) [14]. More importantly, we have found that Th2 responses play a key role in the pathology of lymphedema by promoting fibrosis and inhibiting lymphatic function [16]. In fact, we found that inhibiting Th2 differentiation using monoclonal antibodies directed against interleukin-4 (IL-4) or interleukin-13 (IL-13) markedly decreased fibrosis, leading to resolution of pathologies associated with lymphedema and restoration of lymphatic function [16]. In addition, using a model of lymph node lymphangiogenesis, we found that inhibition of CD4^+^ cells using multiple models (including anti-CD4 monoclonal antibody-mediated depletion, CD4 knockout mice, and blockade of Th2 differentiation with IL-4 or IL-13 monoclonal antibodies) significantly increased lymphangiogenesis in response to inflammation induced by complete Freund’s Adjuvant/ovalbumin immunization [16]. However, while the evidence supporting a role for T cells and Th2 cytokines as negative regulators of lymphatic function is clear, the direct effect of Th2 cytokines on isolated LECs remains poorly understood.

Our previous models of lymphangiogenesis have relied primarily on T cell-mediated inflammatory reactions and expansion of existing lymphatic networks, thereby limiting our understanding of the role of Th2 cytokines in regulating *de novo* lymphangiogenesis. Therefore, the purpose of this study was to determine the effects of Th2 cytokines on isolated LECs and delineate the roles of these mechanisms on *de novo* lymphangiogenesis using the corneal suture model.

## Methods

### Ethics Statement/Animals

This study as well as all procedures and surgeries were approved by the IACUC at Memorial Sloan Kettering Cancer Center. Adult male C57/BL6 mice (10–12 weeks) were purchased from Jackson Labs (Bar Harbor, Maine). Mice were maintained in a light- and temperature-controlled environment, and fed *ad libitum*. Animals were anesthetized using isoflurane. The depth of anesthesia was monitored at least every 15 minutes throughout the procedure by observing that there was no change in respiratory rate associated with surgical manipulation and/or ear, toe, and tail pinch. Analgesia was achieved with subcutaneous injections of Buprenorphine 0.01mg/kg BID to TID. Animals were evaluated three times daily for the first three days following surgery. Animals were examined at least three times weekly thereafter. Animals with any evidence of infection (i.e. periorbital erythema and/or conjunctivitis) or failure to thrive (i.e. decreased feeding and/or activity) were sacrificed. Animals requiring euthanasia were euthanized by carbon dioxide asphyxiation as recommended by the American Veterinary Medical Association (AVMA) Guidelines on Euthanasia.

### Cell culture

Human dermal lymphatic endothelial cells (hLECs) were obtained from PromoCell (Heidelberg, Germany), cultured in ECGM-MV2 media containing 5% FCS, epidermal growth factor (EGF) (5 ng/ml), hydrocortisone (0.2 μg/ml), basic fibroblast growth factor (BFGF) (10 ng/ml), insulin-like growth factor (ILGF) (20 ng/ml), VEGF 165 (0.5 ng/ml), ascorbic acid (1 μg/ml), and penicillin-streptomycin (50 U/ml) (Invitrogen), and passaged every 48 hours. LEC morphology was confirmed with immunofluorescent staining for Prospero homeobox protein 1 (Prox-1) and vascular endothelial factor receptor 3 (VEGFR-3). Experiments were performed using early-passage cells (<10).

### Immunohisto/cytochemistry

Primary antibodies used for chamber slide immunofluorescent staining included Prox-1, Ki67 (both from Abcam, Cambridge, MA), IL-4Rα, and VEGFR-3 (both from R&D Systems, Minneapolis, MN). All secondary antibodies were obtained from Vector Laboratories (Burlingame, CA) and eBioscience (San Diego, CA). Chamber slides were scanned using a Mirax slide scanner (Carl Zeiss, Jena, Germany) and analyzed using Pannoramic Viewer (3D HISTECH, Budapest, Hungary). Signal intensity of Prox-1 was measured using MetaMorph analysis (Molecular Devices, Sunnyvale, CA). Cell counts were performed on high magnification images, with a minimum sample size of 4–6 per group and 4–5 HPF/sample by two blinded reviewers.

### Cellular apoptosis

LEC apoptosis in response to recombinant human (rh) IL-4 or rhIL-13 was quantified using an Annexin V-FITC Apoptosis Detection kit (eBioscience, San Diego, CA) as per the manufacturer’s instructions. Briefly, cells were plated at a seeding density of 9x10^6^ cells/ml and treated with growth media containing 50 ng/ml of rhIL-4 or rhIL-13 protein, or media alone (control). After 24 hours of treatment, cells were harvested, washed and stained with Annexin V-FITC/propidium iodide (PI). Data acquisition was performed using flow cytometry (LSRII;BD Biosciences, San Jose, CA) and analysis was performed using FlowJo software (Tree Star, Ashland, OR). Viable cells were negative for both Annexin V and PI while cells in late stage apoptosis were positive for both Annexin V and PI.

### Cellular proliferation

LEC proliferation was quantified using a Click-iT EdU cell Proliferation Assay Kit (Life Technologies, Grand Island, NY) as per the manufacturer’s instructions. Briefly, cells were plated at a seeding density of 9x10^6^ cells/ml and treated with growth media containing 50 ng/ml of rhIL-4 or rhIL-13 protein, or growth media alone (control). After 24 hours of treatment, a thymidine analogue (EdU, or 5-ethynyl-2’-deoxyuridine) was added directly to the cells in culture, where it was utilized by cycling cells during DNA synthesis. Cells were harvested, fixed, permeabilized, and stained. Data acquisition was performed using flow cytometry (LSRII;BD Biosciences, San Jose, CA) and analysis was performed using FlowJo software (Tree Star, Ashland, OR).

A WST-1 cell assay kit (Cayman Chemical, Ann Arbor, MI) was used according to the manufacturer’s instructions as another method to measure LEC proliferation and confirm our findings with EdU. Briefly, cells were seeded in a 96-well plate at a density of 10^4^–10^5^ cells/well in 100 μl of growth media containing 50 ng/ml of rhIL-4 or rhIL-13 protein, or media alone (control). Cultured cells were placed in an incubator at 37°C for 24 hours. Reconstituted WST-1 mixture was added to each well and then incubated at 37°C for 2 hours. Absorbance of each sample was measured using a microplate reader (Molecular Devices, Sunnyvale, CA) at a wavelength of 450 nm. Finally, the number of actively proliferating LECs was assessed using immunofluorescent chamber slides staining for Ki67 (Abcam, Cambridge, MA).

### Tubule formation assay

The ability of LECs to form connections to nearby cells was analyzed with a matrigel tubule formation assay using a modification of a previously described protocol [17]. Briefly, cells were re-suspended in growth factor-reduced basement membrane matrix (BD Biosciences, San Jose, CA) at a concentration of 1.4×10^5^ cells per 100 μL of matrigel. After thorough mixing, 300 μL of the suspension was added to each well of a 24-well tissue culture plate. The plate was incubated at 37°C for 30 minutes to solidify the matrigel followed by addition of growth media containing 50 ng/ml of rhIL-4, rhIL-13, or media alone (control). Plates were cultured at 37°C and 5% CO_2_ for 12 hours and then imaged using an inverted microscope (Carl Zeiss, Oberkochen, Germany) equipped with MetaMorph scanning and tubule analysis software (Molecular Devices, Sunnyvale, CA).

### Cellular migration assay

To measure *in vitro* LEC migration, we performed a scratch assay as described previously [18]. Briefly, hLECs were plated on a 60-mm dish and then incubated for 6 hours at 37°C to allow cells to adhere and form a confluent monolayer. The cell monolayer was scratched in a straight line with a p200 pipette tip. Debris along the scratch border was removed by washing the cells with 1 ml of growth medium and then replaced with 5 ml of growth media containing 50 ng/ml of either rhIL-4 or rhIL-13 protein, or media containing neither (control). The dishes were maintained at 37°C and 5% CO_2_ for 24 hours. Using a phase-contrast microscope (Nikon, Melville, NY), image acquisition took place at 0, 12, and 24 hours after treatment. Quantitative analysis was performed using Fiji (NIH open-source platform for biological-image analysis).

### Neutralizing antibodies

As previously described, we used monoclonal antibodies against mouse interleukin-4 (IL-4; clone 11B11; 5 μg/g/dose; Bio-X-cell, West Lebanon, NH, USA) or rat antibody against mouse IL-13 (clone 38213; 5 μg/g; R&D Systems, Minneapolis, MN, USA), and administered them intraperitoneally [16]. Controls were treated with similar doses of isotype control antibodies (Bio-X-cell). Mice were treated with IL-4 monoclonal antibody (mAb), IL-13mAb, or isotype control antibodies 24 hours prior to corneal surgery, then weekly (IL-4mAb) or every 4 days (IL-13mAb) for 2 weeks after surgery.

### Mouse model of suture-induced, inflammatory corneal neovascularization

To study the role of IL-4 and IL-13 on LEC proliferation *in vivo*, we used a well-described model of inflammatory corneal neovascularization [19]. Briefly, a 2-mm corneal trephine was gently placed on the central cornea of anesthetized mice solely to mark the central corneal area. Three 11–0 sutures were then placed intrastromally with 2 stromal incursions extending over 120 degrees of corneal circumference each. The outer point of suture placement was chosen as half way between the limbus and the line outlined by the 2-mm trephine, and the inner suture point was equidistant from the 2-mm trephine line to obtain standardized angiogenic responses. Corneal surgeries were performed only on the left eye while the right eye served as an internal control. Sutures were left in place for 14 days and then removed. *In vivo* imaging was then performed using a stereomicroscope (Zeiss, Jena, Germany).

Cornea whole mount staining was performed as previously described [20]. Briefly, corneal tissues were dissected from the rest of the eye tissue using spring scissors under a stereomicroscope and then fixed with 4% (wt/vol) PFA at 4°C overnight. Tissues were then digested with proteinase K (10 mM Tris-HCl buffer, pH 7.4) for 5 min at room temperature, washed in 100% methanol, and then blocked in blocking buffer (3% (wt/vol) milk. Primary antibodies against LYVE-1 (R&D Systems, Minneapolis, MN), PECAM-1 (Santa Cruz Biotechnology, Dallas, TX) and Ki67 (Abcam, Cambridge, MA) were applied and incubated for 16 hours at 4°C on a rocking board. The corneal tissues were then washed and incubated with Alexa Fluor conjugated secondary antibodies (Invitrogen, Eugene OR). Corneal tissues were mounted with VectaMount aqueous mounting medium (Vector Laboratories, Burlingame, CA) after making three slits with a scalpel blade (at respective clockwise direction 120°, 240° and 360°) to flatten out the cornea. Imaging was performed using a Leica SP5-U confocal microscope (Leica Microsystems Inc, Buffalo Grove, IL). Quantification was performed with Imaris software (Bitplane, Zurich, Switzerland).

### Statistical analysis

Statistical analysis was performed using GraphPad Prism software (GraphPad Software, Inc., San Diego, CA). The Student’s T-test was used to compare differences between two groups while ANOVA with *post hoc* tests were used to compare multiple groups. Descriptive analysis and graphical methods were used to analyze and summarize results. Data is presented as mean ± standard deviation unless otherwise noted, with p<0.05 considered significant.

## Results

### **hLECs express IL-4R**α **and respond to rhIL-4/IL-13 with increased apoptosis**

**Fig 1:**
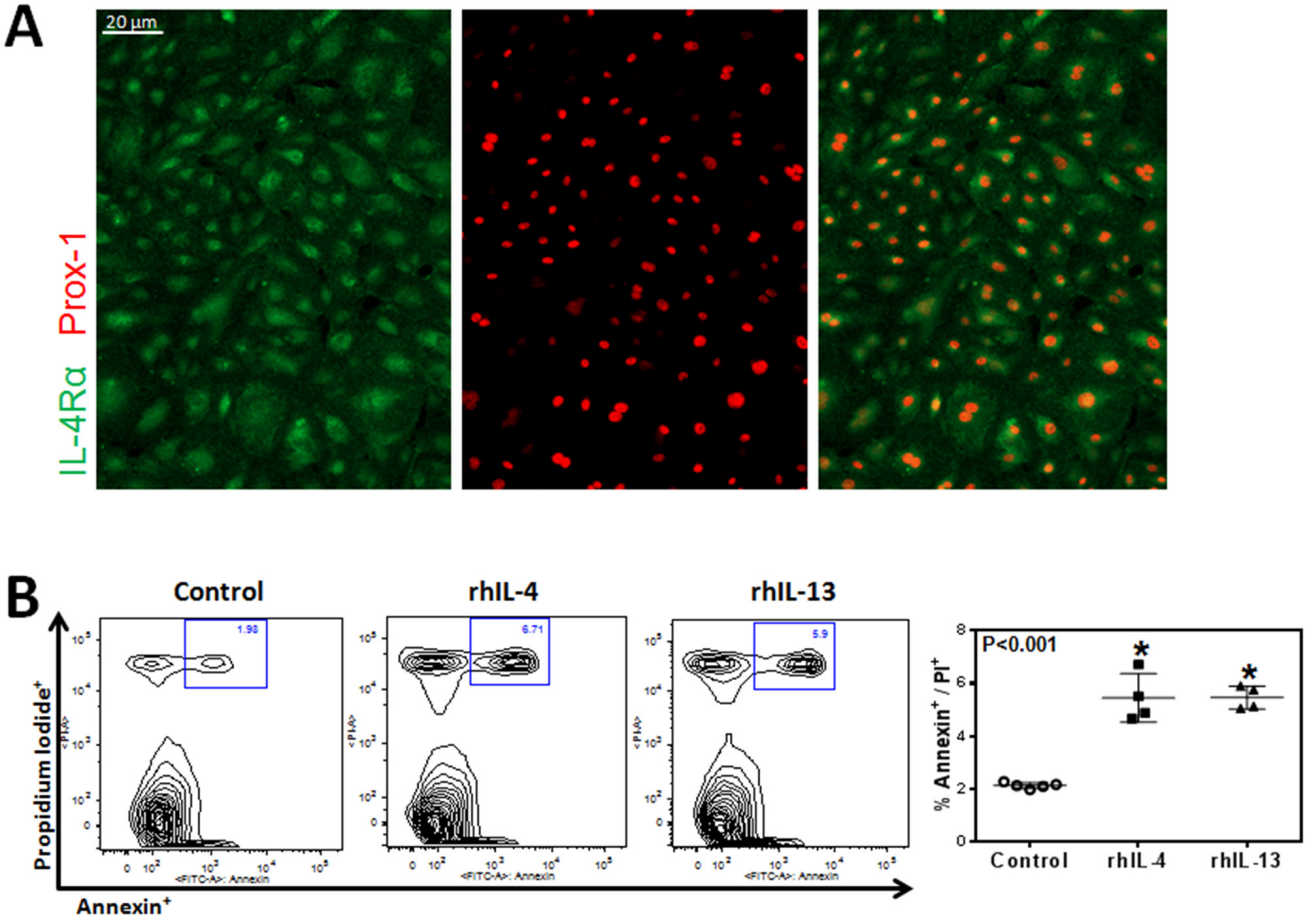
hLECs express IL-4Rα and respond to rhIL-4/IL-13 with increased apoptosis. **A.** Representative immunofluorescent images of IL-4Rα and Prox-1 staining from cultured hLECs. Scale bar = 20μm. **B.** Flow cytometry analysis for Annexin V and propidium iodide of cultured hLECs after treatment with 50 ng/ml rhIL-4 or rhIL-13 protein, or media alone (control) for 24 hours. Representative contour plots. Quantification of apoptotic hLECs. Control vs. rhIL-4 (n = 5 in each; *p<0.001); control vs. rhIL-13 (n = 5 in each; *p<0.001); rhIL-4 vs. rhIL-13 (n = 5 in each; p = NS).

To examine the effect of IL-4 and IL-13 on LECs, we first sought to determine whether LECs express the IL4R*α*, a cognate receptor for IL-4 and IL-13. Immunofluorescent staining confirmed expression of IL-4R*α*, the common signaling receptor for IL-4 and IL-13 on hLECs (Fig 1A). This confirmed that LECs can respond to IL-4 and IL-13. In addition, we found that 24 hour incubation of LECs with rhIL-4 or rhIL-13 significantly (more than 2x) increased apoptosis as reflected by increased expression of Annexin V (Fig 1B; P<0.001 for both).

### rhIL-4 and rhIL-13 decrease LEC proliferation and downregulate expression of Prox-1

**Fig 2:**
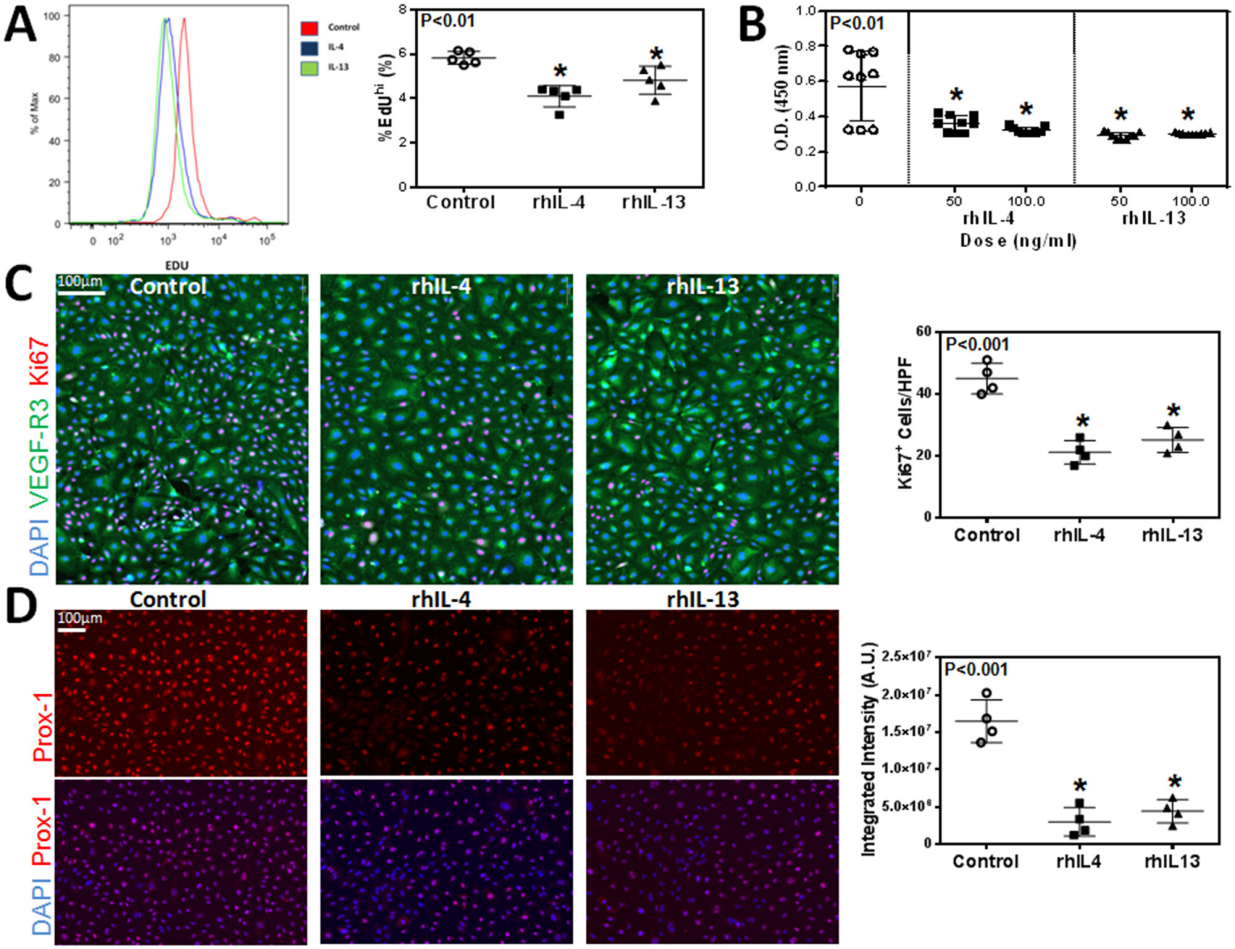
rhIL-4 and rhIL-13 decrease LEC proliferation and downregulate expression of Prox-1. **A.** Flow cytometry analysis for incorporated EdU by cultured hLECs after treatment with 50 ng/ml rhIL-4 or rhIL-13 protein, or media alone (control) for 24 hours. Representative histograms. Quantification of EdU positive hLECs. Control vs. rhIL-4 (n = 5 in each; *p<0.01); control vs. rhIL-13 (n = 5 in each; *p<0.01); rhIL-4 vs. rhIL-13 (n = 5 in each; p = NS). **B.** Quantification of hLEC proliferation using a WST-1 cell assay kit. Control vs. rhIL-4 (n = 10 in each; *p<0.01 for both concentrations); 50 ng/ml rhIL-4 vs. 100 ng/ml rhIL-4 (n = 10 in each; *p<0.05); control vs. rhIL-13 (n = 10 in each; *p<0.01 for both concentrations); 50 ng/ml rhIL-13 vs. 100 ng/ml rhIL-13 (n = 10 in each; p = NS). **C.** Representative immunofluorescent staining for Ki67 and VEGF-R3 in cultured hLECs after treatment with 50 ng/ml rhIL-4 or rhIL-13 protein, or media alone (control) for 24 hours. Scale bar = 100μm. Quantification of Ki67^+^ cells per high-power field. Control vs. rhIL-4 (n = 5 in each; *p<0.001); control vs. rhIL-13 (n = 5 in each; *p<0.001); rhIL-4 vs. rhIL-13 (n = 5 in each; p = NS). **D.** Representative immunofluorescent staining for Prox-1 in cultured hLECs after treatment with 50 ng/ml rhIL-4 or rhIL-13 protein, or media alone (control) for 24 hours. Scale bar = 100μm. Quantification of Prox-1 signal intensity using MetaMorph analysis per high-power field. Control vs. rhIL-4 (n = 5 in each; *p<0.001); control vs. rhIL-13 (n = 5 in each; *p<0.001); rhIL-4 vs. rhIL-13 (n = 5 in each; p = NS).

Given the effect of rhIL-4 and rhIL-13 on hLEC apoptosis, we next investigated their effect on proliferation and found that treatment with rhIL-4 or rhIL-13 markedly decreased incorporation of EdU by cultured LECs (Fig 2A; P<0.01 for both). These findings were confirmed using a WST-1 assay by analyzing the number of metabolically active cells. This demonstrated that treatment with either rhIL-4 or rhIL-13 significantly decreased cellular proliferation as compared with controls (Fig 2B; P<0.01 for both). This effect was dose-dependent with rhIL-4, while the inhibitory effects of rhIL-13 were maximal even at the lowest dose analyzed. In addition, we found that treatment with either rhIL-4 or rhIL-13 significantly decreased the number of cells that stained positive for Ki67, a marker of cellular proliferation (Fig 2C; P<0.001 for both). Interestingly, we found that treatment with rhIL-4 or rhIL-13 markedly decreased the expression of Prox-1 by LECs. As expected, we found that control cells had high, uniform levels of Prox-1 staining; in contrast, treatment with rhIL-4 or rhIL-13 resulted in a more than 3-fold decrease in Prox-1 expression using immunocytochemistry evidence (Fig 2D; P<0.001 for both).

### rhIL-4 and rhIL-13 inhibit hLEC tubule formation and migration

**Fig 3:**
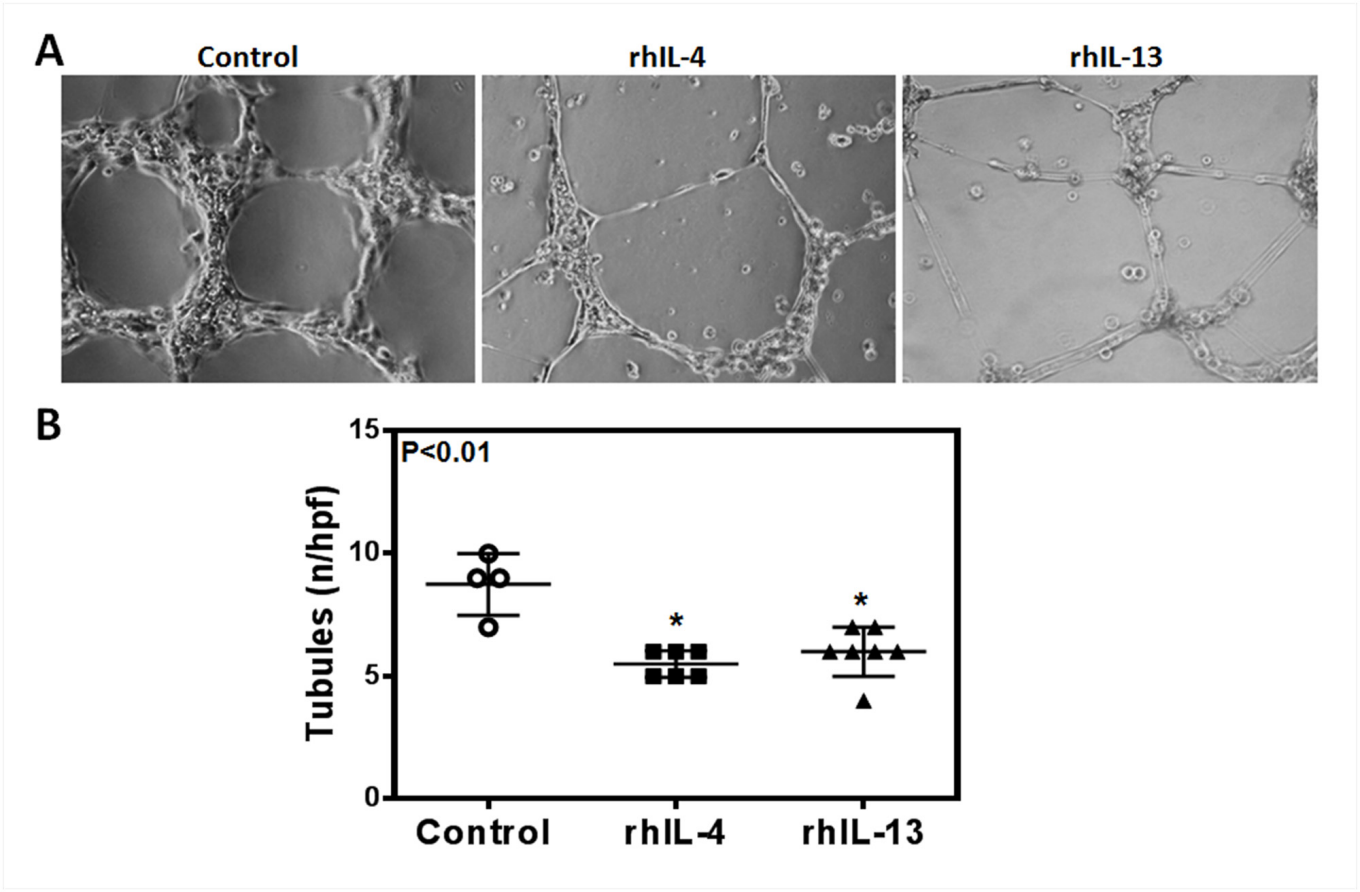
rhIL-4 and rhIL-13 inhibit hLEC tubule formation. **A.** Representative images of cultured hLECs tubule formation on matrigel after treatment with 50 ng/ml rhIL-4 or rhIL-13 protein, or media alone (control) for 12 hours. **B.** Quantification of tubule formation per high-power field. Control vs. rhIL-4 (n = 5–8 in each; *p<0.01); control vs. rhIL-13 (n = 5–8 in each; *p<0.01); rhIL-4 vs. rhIL-13 (n = 5–8 in each; p = NS).

**Fig 4:**
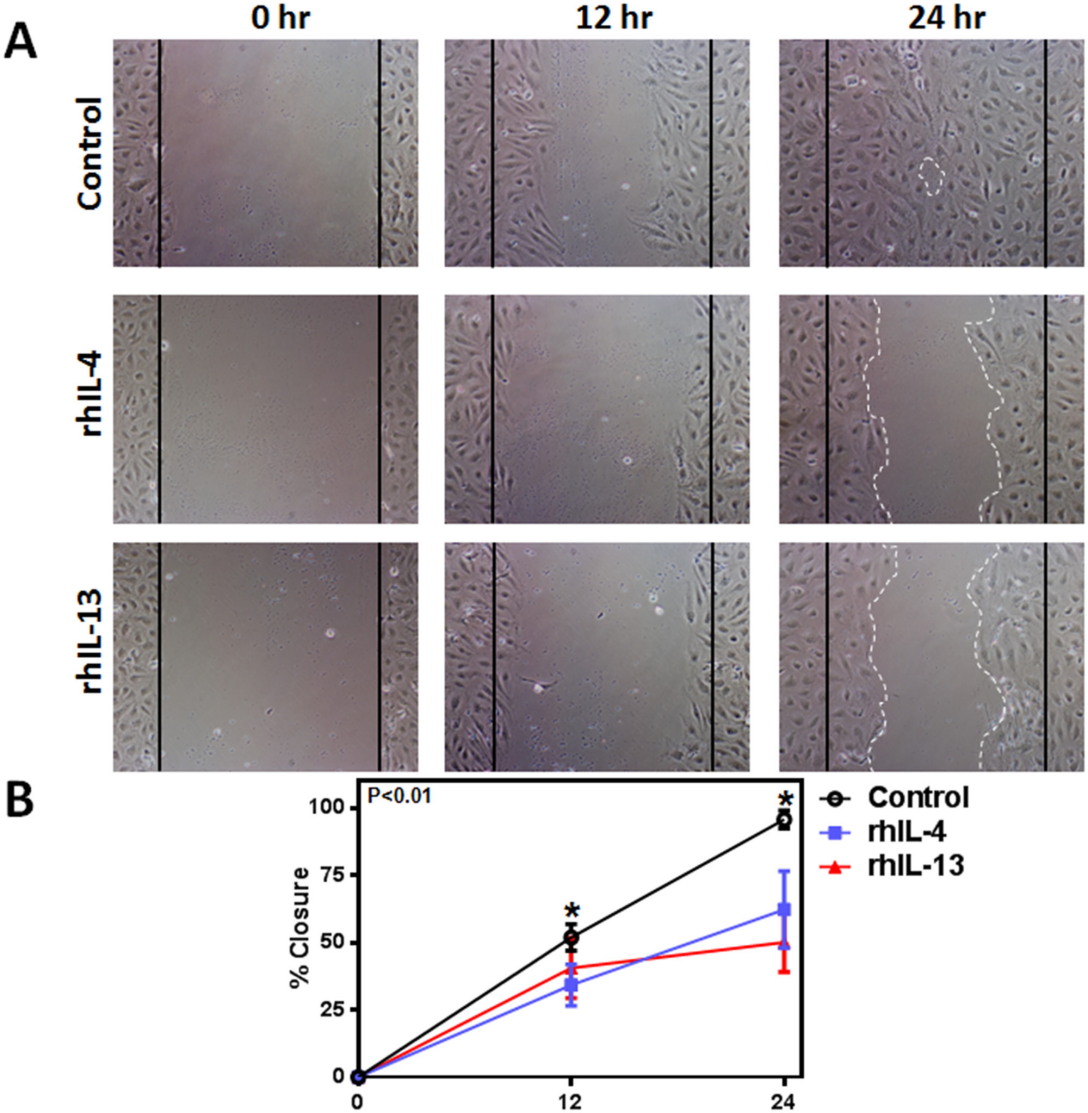
rhIL-4 and rhIL-13 inhibit hLEC migration. **A.** Representative images of cultured hLECs after scratch wound and treatment with 50 ng/ml rhIL-4 or rhIL-13 protein, or media alone (control) at 0, 12 and 24 hours. White dotted lines represent LEC migration borders. Black solid lines represent initial gap created by scratch. **B.** Quantification of hLEC migration at 12 and 24 hours. Control vs. rhIL-4 (n = 5 in each; *p<0.01 for both time points); control vs. rhIL-13 (n = 5 in each; *p<0.01 for both time points); rhIL-4 vs. rhIL-13 (n = 5 in each; p = NS for both time points).

To determine the effect of rhIL-4 and rhIL-13 on the ability of LECs to form connections to nearby cells, we performed a matrigel tubule formation assay. 12 hours after treatment with rhIL-4 or rhIL-13, tubule formation was not only significantly diminished, but was also more erratic in the treatment groups compared to controls (Fig 3A and 3B; P<0.01 for both). These changes correlated with a significant decrease in LECs’ ability to migrate after treatment with rhIL-4 or rhIL-13 in a scratch wound assay (Fig 4A and 4B; P<0.01 for both). Thus, while untreated cells almost completely filled the gap by 24 hours, cells treated with rhIL-4 or rhIL-13 remained widely spaced apart, suggesting that this treatment decreases LEC migration.

### IL-4 and IL-13 blockade promotes lymphangiogenesis but not angiogenesis

**Fig 5:**
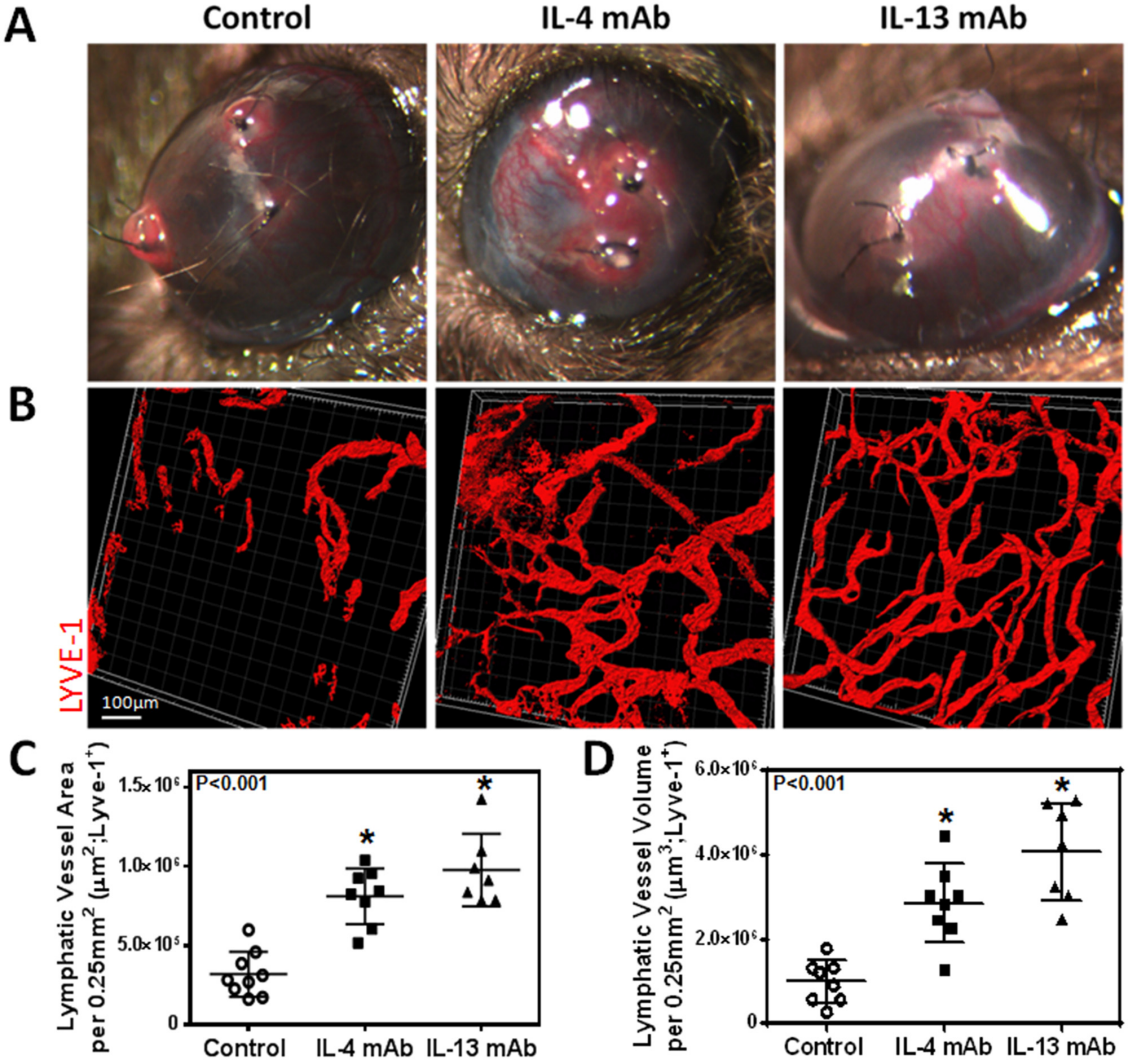
IL-4 or IL-13 blockade increases inflammatory lymphangiogenesis. **A.** Representative gross images of corneas 14 days after suture placement. **B.** Representative immunofluorescent cornea whole mount images for LYVE-1. Scale bar = 100μm. **C.** Quantification of lymphatic vessel area per 0.25mm^2^. Control vs. IL-4mAb (n = 8 in each; *p<0.001); control vs. IL-13mAb (n = 8 in each; *p<0.001); IL-4mAb vs. IL-13mAb (n = 8 in each; p = NS). **D.** Quantification of lymphatic vessel volume per 0.25mm^2^. Control vs. IL-4mAb (n = 8 in each; *p<0.001); control vs. IL-13mAb (n = 8 in each; *p<0.001); IL-4mAb vs. IL-13mAb (n = 8 in each; *p<0.05).

**Fig 6:**
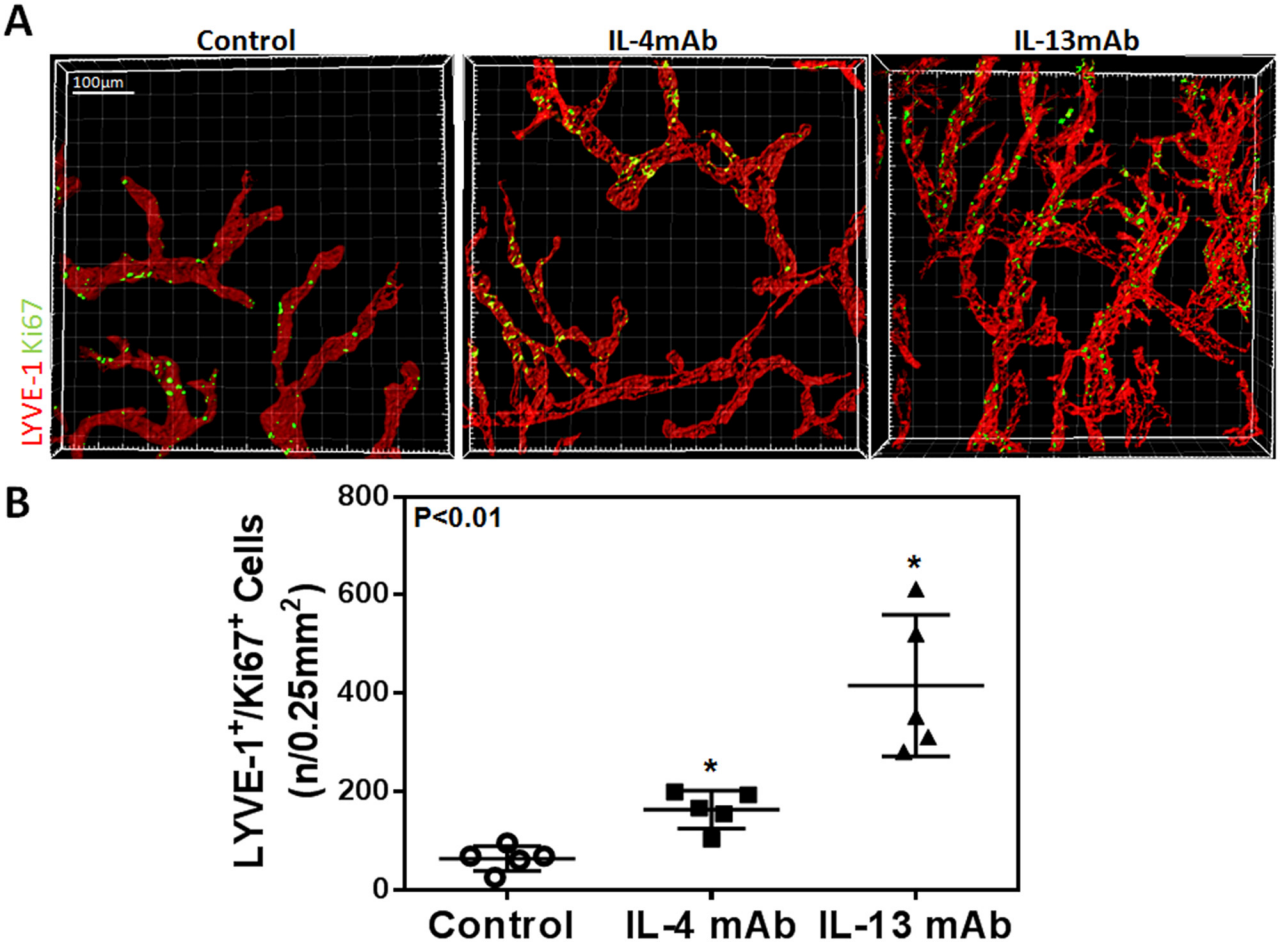
IL-4 and IL-13 blockade increases LEC proliferation *in vivo*. **A.** Representative immunofluorescent cornea whole mount images stained for Ki67 and LYVE-1. Scale bar = 100μm. **B.** Quantification of Ki67^+^/LYVE-1^+^ cells (n/0.25mm^2^). Control vs. IL-4mAb (n = 5 in each; *p<0.01); control vs. IL-13mAb (n = 5 in each; *p<0.01); IL-4mAb vs. IL-13mAb (n = 5 in each; *p<0.01).

**Fig 7:**
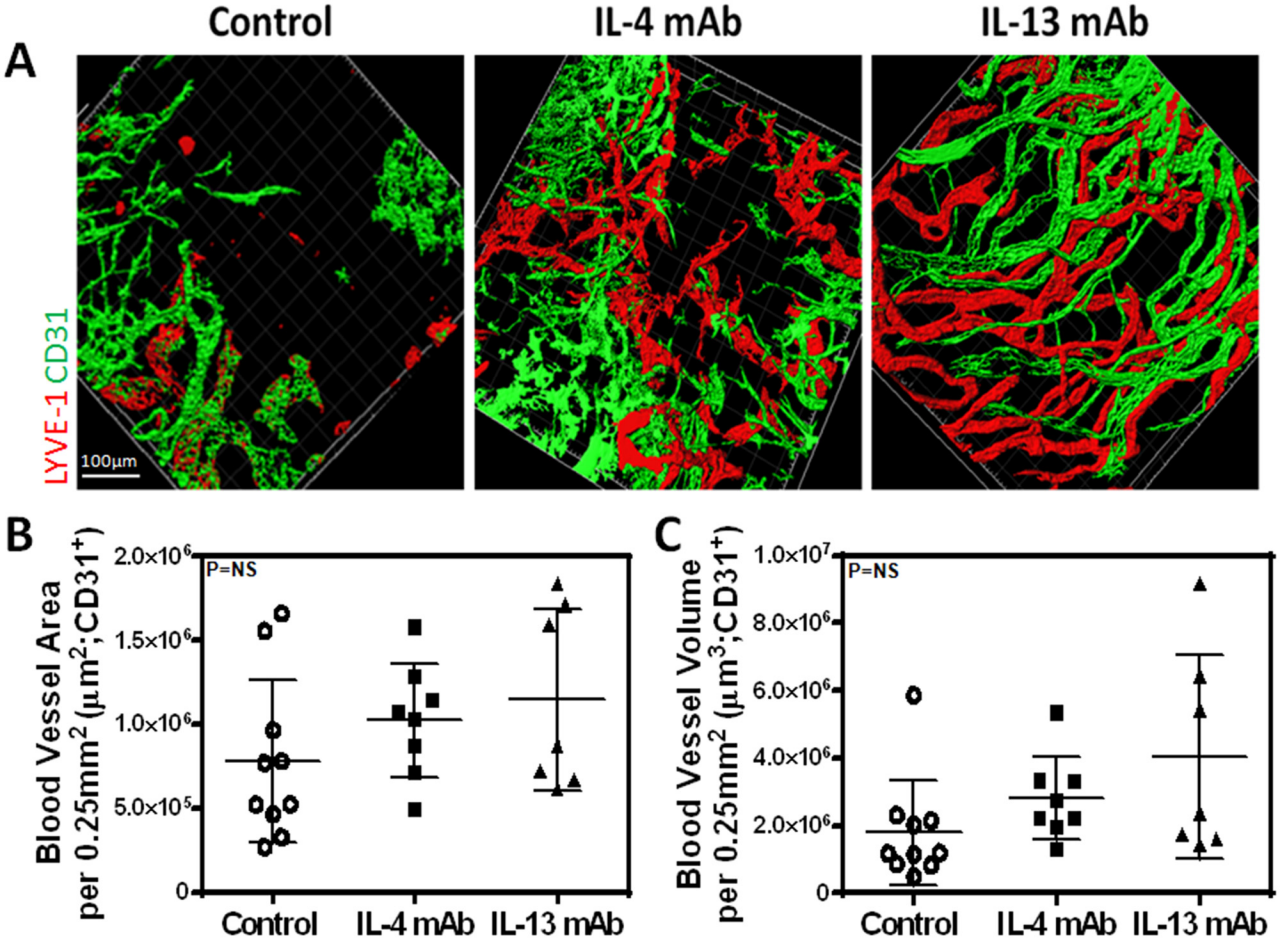
IL-4 and IL-13 blockade does not increase corneal angiogenesis. **A.** Representative immunofluorescent cornea whole mount images stained for CD31 and LYVE-1. Scale bar = 100μm. **B.** Quantification of blood vessel area per 0.25mm^2^. Control vs. IL-4mAb (n = 8 in each; p = NS); control vs. IL-13mAb (n = 8 in each; p = NS); IL-4mAb vs. IL-13mAb (n = 8 in each; p = NS). **C.** Quantification of blood vessel volume per 0.25mm^2^. Control vs. IL-4mAb (n = 8 in each; p = NS); control vs. IL-13mAb (n = 8 in each; p = NS); IL-4mAb vs. IL-13mAb (n = 8 in each; p = NS).

Given our *in vitro* findings, we next hypothesized that blocking IL-4 and IL-13 using neutralizing antibodies would increase lymphangiogenesis *in vivo*. Two weeks after suture placement and treatment with IL-4mab and IL-13mab, we performed lymphatic vessel analysis using LYVE-1 whole mount staining which demonstrated a marked increase in lymphangiogenesis in animals treated with IL4-mab or IL-13mab as compared with controls (Fig 5B, 5C and 5D; P<0.001 for both). Consistent with our *in vitro* findings, we noted that neutralization of IL-4 or IL-13 markedly increased LEC proliferation as reflected by increased expression of Ki67 by LYVE-1 positive LECs (Fig 6A and 6B; P<0.01 for both). Interestingly, IL-13 blockade was more potent in promoting lymphangiogenesis as compared with IL-4. In contrast, we found no significant differences in angiogenesis (Fig 7A, 7B and 7C; P = NS for both). In conclusion, not only did blockade of these cytokines promote LEC proliferation compared to controls, but blockade of IL-13 had a greater effect on LEC proliferation than IL-4 blockade.

## Discussion

In the current study, we found that Th2 cytokines, IL-4 and IL-13, have profound anti-lymphangiogenic effects by increasing LEC apoptosis and inhibiting proliferation, tubule formation, and migration both *in vitro* and *in vivo*. These findings are important since we have previously shown that the expression of Th2 cytokines is markedly increased in lymphedema [16]. Our previous work provided substantial evidence that Th2 cytokines inhibit lymphatic function by increasing fibrosis and promoting changes in the extracellular matrix. Our current work suggests that in addition to these effects, Th2 cytokines may impair lymphatic repair and regeneration via direct effects on LECs.

A large number of studies have shown that lymphangiogenesis is regulated by the expression of potent pro-lymphangiogenic molecules, including members of the VEGF family, hepatocyte growth factor, and fibroblast growth factor, among others. These cytokines and growth factors promote lymphangiogenesis via diverse effects on LECs by regulating cellular proliferation, migration, tubule formation, and survival. However, more recent studies have suggested that this process is balanced by potent anti-lymphangiogenic mechanisms [2, 8, 11]. For example, Oka *et al*. and Clavin *et al*. reported that TGF-β1, independent of VEGF-C, impairs lymphangiogenesis and downregulates expression of lymphatic specific genes such as VEGF-R3 and Prox-1 [7, 8]. More recently, Kataru *et al*. demonstrated that T cell-derived IFNγ potently impairs inflammatory lymph node lymphangiogenesis [11]. Thus, the balance between pro- and anti-lymphangiogenic mechanisms is a key lymphatic function regulatory mechanism.

A number of studies suggest that these anti-lymphangiogenic mechanisms can, in some circumstances, override the effects of pro-lymphangiogenic molecules. For example, Rutkowski *et al*. showed that lymphatic function was markedly impaired in mice with tail lymphatic injury despite increased VEGF-C expression and lymphatic vessel hyperplasia [21]. These findings were corroborated by Goldman *et al*. using the same model demonstrating that even supra-physiological doses of VEGF-C failed to improve lymphatic regeneration and function [22]. More recently, our group has shown that the expression of potent anti-lymphangiogenic cytokines, such as TGF-β1, IFNγ, and endostatin, are also markedly increased in this setting [6, 14]. In addition, the inhibition of these pathways, independent of VEGF-C expression, potently increases lymphatic regeneration and function [9].

Our current study provides substantial evidence that Th2 cytokines, similar to IFNγ and TGF-β1, also have profound anti-lymphangiogenic effects. These findings are consistent with a recent study demonstrating that Th2 cytokines regulate LEC proliferation and gene expression, and that inhibition of these pathways in an asthma model increases lymphangiogenesis and improves pulmonary function [23]. In addition, we show that the anti-lymphangiogenic effects of IL-4 and IL-13 occurred despite the presence of lymphangiogenic growth factors (VEGF, FGF, ILGF, EGF) found in the *in vitro* endothelial cell growth medium. This provides similar evidence to previous studies regarding other anti-lymphangiogenic cytokines, such as TGF-β1, that act independently of pro-lymphangiogenic growth factors [7, 8]. More specifically, anti-lymphangiogenic cytokines, such as TGF-β1 and IFNγ, act by downregulating Prox-1 expression in LECs [7, 11]. Our findings suggest that IL-4 and IL-13 also downregulate Prox-1 expression in LECs, indicating that this may also be the underlying mechanism as to how IL-4 and IL-13 function as anti-lymphangiogenic cytokines. Taken together, these findings suggest that inhibition of Th2 cytokines in lymphedema may represent a novel means of improving lymphatic function and decreasing the pathology of the disease without the need for exogenous delivery of VEGF-C or other pro-lymphangiogenic molecules.

In conclusion, using *in vitro* and *in vivo* studies we have shown that IL-4 and IL-13 administration in physiologic doses have profound anti-lymphangiogenic effects and potently impair LEC survival, proliferation, migration, and tubule formation. Inhibition of these cytokines using targeted monoclonal antibodies specifically increases inflammatory lymphangiogenesis without concomitant changes in angiogenesis. These findings, together with recent studies from our group and others, suggest that manipulation of anti-lymphangiogenic pathways *in vivo* may represent a novel and potent means of improving lymphangiogenesis.
